# Grain Size Influence on the Magnetic Property Deterioration of Blanked Non-Oriented Electrical Steels

**DOI:** 10.3390/ma14227055

**Published:** 2021-11-20

**Authors:** Lucas Boehm, Christoph Hartmann, Ines Gilch, Anett Stoecker, Rudolf Kawalla, Xuefei Wei, Gerhard Hirt, Martin Heller, Sandra Korte-Kerzel, Nora Leuning, Kay Hameyer, Wolfram Volk

**Affiliations:** 1 Chair of Metal Forming and Casting (utg), Technical University of Munich, 85748 Garching, Germany; christoph.hartmann@utg.de (C.H.); ines.gilch@utg.de (I.G.); wolfram.volk@utg.de (W.V.); 2 Institute of Metal Forming (IMF), TU Bergakademie Freiberg, 09596 Freiberg, Germany; anett.stoecker@imf.tu-freiberg.de (A.S.); rudolf.kawalla@imf.tu-freiberg.de (R.K.); 3 Institute of Metal Forming (IBF), RWTH Aachen University, 52056 Aachen, Germany; xuefei.wei@ibf.rwth-aachen.de (X.W.); gerhard.hirt@ibf.rwth-aachen.de (G.H.); 4 Institute for Physical Metallurgy and Materials Physics (IMM), RWTH Aachen University, 52074 Aachen, Germany; heller@imm.rwth-aachen.de (M.H.); Korte-Kerzel@imm.rwth-aachen.de (S.K.-K.); 5 Institute of Electrical Machines (IEM), RWTH Aachen University, 52062 Aachen, Germany; nora.leuning@iem.rwth-aachen.de (N.L.); kay.hameyer@iem.rwth-aachen.de (K.H.)

**Keywords:** grain size, blanking, electrical steel, magnetic properties

## Abstract

Non-oriented electrical steel sheets are applied as a core material in rotors and stators of electric machines in order to guide and magnify their magnetic flux density. Their contouring is often realized in a blanking process step, which results in plastic deformation of the cut edges and thus deteriorates the magnetic properties of the base material. This work evaluates the influence of the material’s grain size on its iron losses after the blanking process. Samples for the single sheet test were blanked at different cutting clearances (15 µm–70 µm) from sheets with identical chemical composition (3.2 wt.% Si) but varying average grain size (28 µm–210 µm) and thickness (0.25 mm and 0.5 mm). Additionally, in situ measurements of blanking force and punch travel were carried out. Results show that blanking-related iron losses either increase for 0.25 mm thick sheets or decrease for 0.5 mm thick sheets with increasing grain size. Although this is partly in contradiction to previous research, it can be explained by the interplay of dislocation annihilation and transgranular fracturing. The paper thus contributes to a deeper understanding of the blanking process of coarse-grained, thin electrical steel sheets.

## 1. Introduction

Soft magnetic cores of high permeability are used in electric drives in order to amplify magnetic flux densities. These magnetic cores mostly consist of stacked, insulated sheets of non-oriented electrical steel with alloying contents of silicon (up to 3.2%) and aluminum (1%) and sheet thicknesses between 0.1–0.5 mm. Soft magnetic properties are based on domain wall displacement, and the quality of electrical steels is characterized by their loss per cycle in W/kg at a certain frequency and induction [1].

Various authors have evaluated the influence of laser cutting, blanking and spark erosion on the magnetic properties of electrical steel [2,3,4]. Due to its cost effectiveness at high production volumes, blanking is the most widespread cutting process for electrical steel sheets in the production of electric drives.

However, plastic deformation of the cut edges introduces dislocations surrounded by microstress, which effectively hinders domain wall motion and domain rotation. As a consequence, this leads to lower permeability, larger coercivity and larger hysteresis losses. Moreover, stresses in electrical steel affect the magnetic properties as a result of the inverse magnetostrictive effect [5].

It was shown that residual stresses after blanking reach further into the material than the strain-hardened area [6]. Neutron grating interferometry measurements demonstrated that the magnetic properties of blanked sheets are affected at distances of up to three times the blank thickness away from the cut edge [7]. LoBue et al. [8] showed that stresses in electrical steel actually increase the total losses and that compressive stress aggravates the total losses much more than tensile stress. Other studies also proved the negative influence of compressive stress [9,10]. Low levels of tensile stress, however, can also reduce losses [10]. According to Weiss [11], the blanking work until the blanking force maximum is suitable for the assessment of magnetic property deterioration by increasing cutting clearances and tool wear, because it correlates with the amount of plastically deformed material volume next to the blanked edge. However, there are ways to reduce the blanking influence. Blanking-related iron losses are reduced by the use of thinner steel sheets [11,12]. Furthermore, the use of small cutting clearances [13,14] and sharp tools [14] can reduce iron losses in blanked electrical steel sheets. Lower alloying contents of silicon reduce yield strength and tensile strength of electrical steels and thus reduce the material volume affected by blanking [7]. Lower cutting speeds also minimize losses [6]. A peculiarity of non-oriented electrical steel sheets is their large grain size of up to 200 µm [15]. A combination of large grain sizes, small sheet thicknesses and small cutting clearances leads to the blanking process being prone to size effects. According to Vollertsen [16], ‘Size effects are deviations from intensive or proportional extrapolated extensive values of a process which occur, when scaling the geometrical dimensions’. In blanking experiments on CuZn15 sheets, the shear strength is increased for a combination of large grain sizes, small sheet thicknesses and small cutting clearances [17]. The shear strength is proportional to the blanking work.

Kuo et al. [13] compared two electrical steel grades with almost identical magnetic behavior at 50 Hz but different chemical composition and grain size. From blanking experiments, the authors concluded that electrical steels with smaller grain sizes suffer from less magnetic property deterioration by blanking. Their explanation is twofold. Firstly, they concluded that a small-grained material has a higher dislocation density and thus a better deformability, which results in less residual stress after blanking. Secondly, the small-grained material is supposed to be less sensitive to residual stresses, as its higher grain boundary density impedes domain wall motion.

In this work, the influence of grain size on the magnetic properties after blanking is further evaluated. Different grain sizes were realized in steel sheets of identical chemical composition by variations of the production process. The objective of this work is to contribute to the understanding of grain size effects on the magnetic properties of electrical steel after blanking.

## 2. Materials and Methods

The electrical steel sheets that were produced and evaluated for this work originated from the DFG financed research group FOR 1897 ‘Low-Loss Electrical Steel Sheets for Energy-Efficient Electrical Drives’. The aim of the research group is to analyze the whole production process of electrical steel parts from slabs to blanked sheets, starting with an electrical steel slab with 3.2 wt.% silicon content and 34 mm thickness. The chemical composition of the material was measured by optical emission spectroscopy and is given in Table 1.

The steel slab was hot rolled to hot strips of 1 mm thickness. A rolling finishing temperature of 850 °C followed by water quenching lead to a banded microstructure, while a rolling finishing temperature of 880 °C with subsequent furnace cooling to room temperature at 50 K/h resulted in a fully recrystallized, homogeneous microstructure.

In this work, only material with a homogeneous hotband structure is evaluated, since this is the dominating case in industry applications. Subsequent cold rolling produced cold strips of 0.5 mm and 0.25 mm thickness. Afterwards, the steel sheets were annealed in a customized annealing furnace at either 900 °C for 120 s, 1000 °C for 60 s or 1100 °C for 60 s.

**Figure 1:**
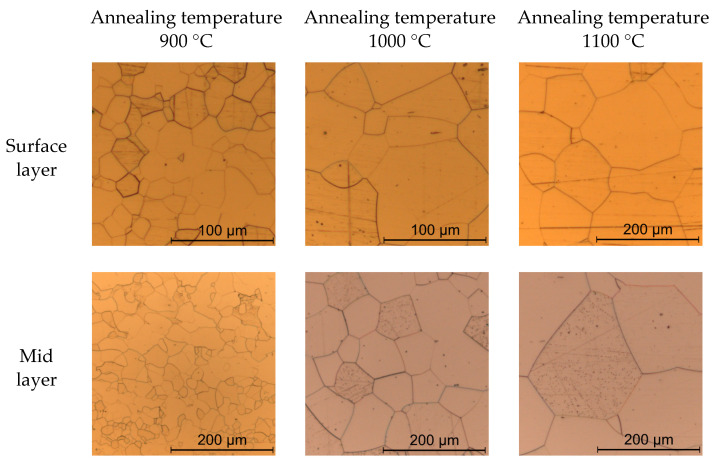
Microstructure images of steel sheets with 0.5 mm sheet thickness.

**Figure 2:**
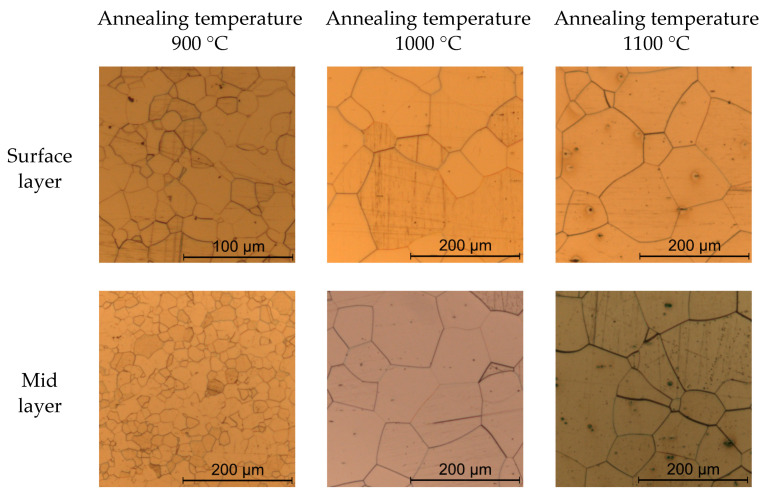
Microstructure images of steel sheets with 0.25 mm sheet thickness.

**Figure 3:**
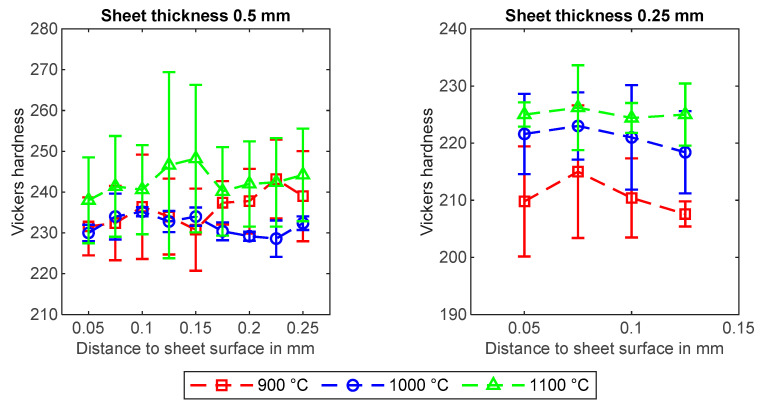
Results of hardness measurements on the steel sheets’ cross sections. A Vickers spike and a test force of 0.25 N were used for the measurements.

During the annealing process, strip tension was applied to the cold rolled sheets so as to mimic an industrial continuous heat treatment process where coiling creates the strip tension. A strip tension of around 80% yield stress, under consideration of the respective annealing temperatures, resulted in minimum buckling of the sheets. The atmosphere in the annealing furnace was static ambient air of unknown humidity. To prevent oxidation, the steel sheets were wrapped in protective foil. The annealing process determined the final grain size and texture of the different steel sheets. Hot rolling, cold rolling and annealing resulted in six different types of electrical steel sheets with distinct average grain sizes. The hot rolling, cold rolling and annealing process for the investigated materials is described in detail in [18]. Grain size measurement by the line intercept method in the rolling direction and the transverse direction was realized in the surface plane of the sheets at around 90% sheet thickness and in the mid layer at around 50% sheet thickness. Therefore, material samples were ground and polished with a 1 µm diamond suspension and an ethanol-based lubricant. To enhance the visibility of grain boundaries, material samples were etched with Nital 5%. The average grain size was calculated, considering at least 300 individual grains per layer and orientation to rolling direction. The differences in grain size between the mid-layer and surface layer are due to changing deformation structures across the sheet thickness [19]. Table 2 shows an overview of the produced electrical steel sheets, and Figure 1 and Figure 2 show light microscopy images of their microstructures. There was no visible oxide layer on the sheets in light microscopy images of the sheets’ cross sections. Nevertheless, hardness measurements were carried out in order to check for the possibility of a thin oxide layer on the steel sheets, which might affect blanking. The idea is that if there was decarburization of the steel sheets during the annealing process, the hardness of the material close to the surface must be reduced. Figure 3 shows the results of hardness measurements for the investigated materials. Each data point represents five individual hardness measurements at the same distance to the sheet surface. Error bars show the standard deviation. Table 2 shows the total Vickers hardnesses of the different materials. The hardness measurements show no decrease in Vickers hardness towards the sheet surface. Decarburization of the steel sheets as a consequence of annealing cannot be fully excluded; however, it is not measurable.

The blanking of electrical steel strips and their preparation for the single sheet test was done with the same blanking tool as in, and according to [14]. This tool allows for the blanking of electrical steel strips with a size of 5 × 60 mm^2^ or 60 × 60 mm^2^. Throughout the blanking experiments, the cutting line was perpendicular to the rolling direction of the steel sheets. The tool can further be equipped with different dies to realize increasing cutting clearances. Table 3 shows which cutting clearances were used for different sheet thicknesses, as well as the corresponding relative cutting clearance in percent of the sheet thickness.

A single sheet tester with a square measuring room of 60 × 60 mm^2^ was applied for the evaluation of iron losses in the blanked samples. Square samples with an edge length of 60 mm served as reference, representing the uncut material. For the 0.25 mm thick sheets, the reference samples were blanked with a cutting clearance of 15 µm, and for the 0.5 mm thick sheets the reference samples were blanked with a cutting clearance of 35 µm. The blanking effect on the reference samples is considered very low. On the other hand, by inserting 12 strips of 5 mm width into the single sheet tester, the blanking-affected material volume is 12 times higher. Therefore, a comparison of the iron losses between the single square reference sample and the composed twelve 5 × 60 mm^2^ strips reveals the blanking influence. Hysteresis curves and iron losses were measured under a sinusoidal magnetic flux excitation, a maximum polarization of 1 T and at a rather low excitation frequency of 50 Hz. This is because the influence of blanking-related losses is particularly pronounced for lower frequencies due to the reduction of eddy currents [20]. The magnetization direction was parallel to the cutting line throughout all measurements. Following the findings of Weiss [11], the punch force over punch travel curve was measured, and the blanking work until the blanking force maximum was calculated. The measurement was done according to the description in [14] and in order to draw conclusions on the magnetic property degradation after blanking.

## 3. Results

Considering the two thicknesses after cold rolling, three annealing temperatures and three relative cutting clearances, a total of 18 different samples were prepared for the single sheet test, each one consisting of 12 individual 5 × 60 mm^2^ blanked strips. In the following, blanking work and iron losses are correlated to the average grain size in the mid layer of the sheets, as it is considered to be representative for the whole sheet, despite the fact that grain size on the sheet surface was generally lower.

### 3.1. Blanking Work

**Figure 4:**
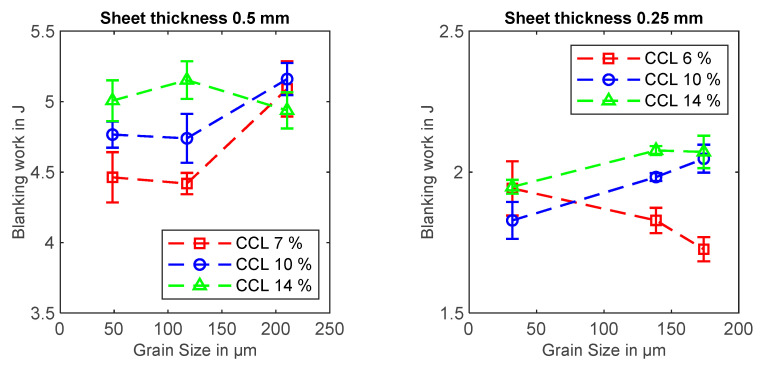
Blanking work in dependence of the average grain size for sheet thicknesses of 0.25 mm or 0.5 mm and for cutting clearances of 6%, 7%, 10% and 14%. Error bars show the standard deviation of the blanking work.

During the blanking process, the punch force over punch travel curve was measured for the first five blanking operations of each parameter combination. The results of the blanking work measurements are displayed in Figure 4. For both sheet thicknesses, the blanking work is mostly increasing with increasing grain size. However, for 0.25 mm thick sheets blanked with 6% cutting clearance, the blanking work is clearly decreasing with increasing grain size. Furthermore, there is a tendency towards increasing blanking work with increasing cutting clearance.

### 3.2. Iron Losses

**Figure 5:**
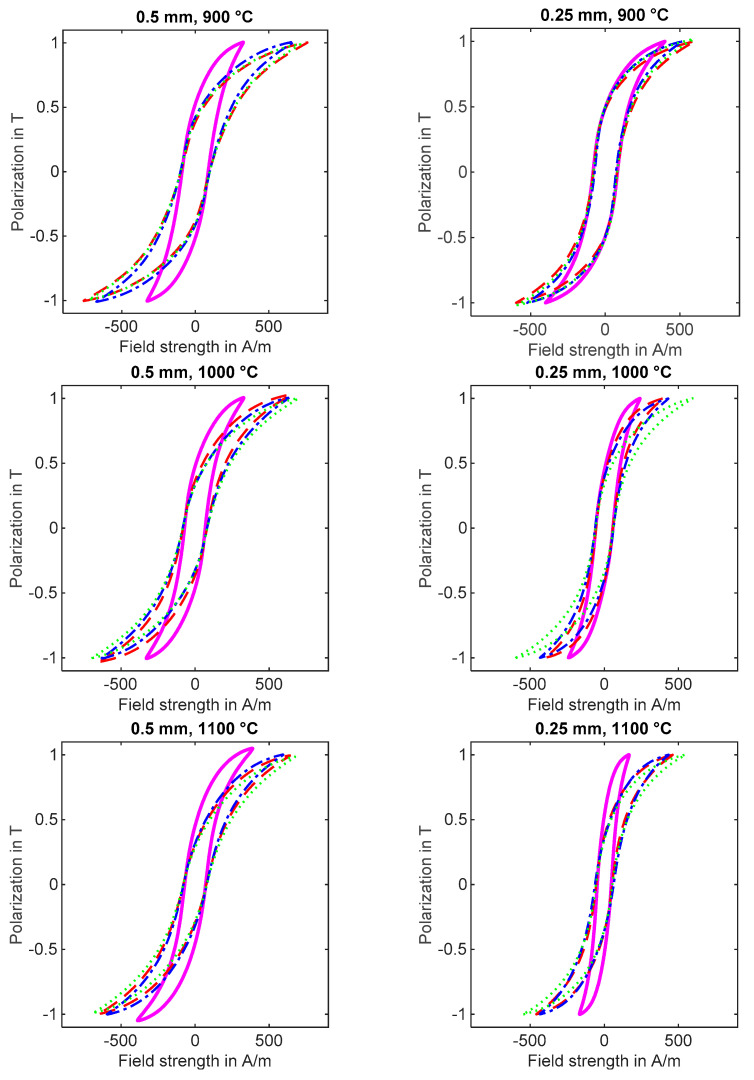

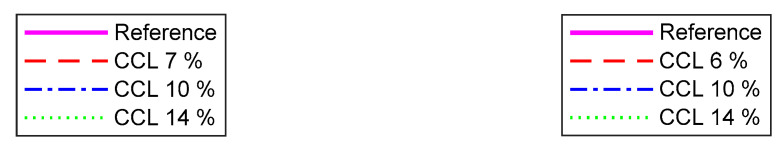
Hysteresis loops for square reference samples and composed strip samples at a frequency of 50 Hz. Left column: sheet thickness of 0.5 mm and annealing temperatures of 900 °C, 1000 °C and 1100 °C, respectively. Cutting clearance was 7%, 10% and 14%. Right column: sheet thickness of 0.25 mm and annealing temperatures of 900 °C, 1000 °C and 1100 °C. Cutting clearance was 6%, 10% and 14%.

Iron losses were calculated based on hysteresis measurements for the square reference samples and the composed blanked samples. The results of the hysteresis measurements are displayed in Figure 5. More information on the magnetic properties of the evaluated materials can be found in [21]. In Equation (1), a specific iron loss factor C is introduced according to [14] in order to facilitate the comparison of blanking-related iron losses between differently processed samples. The iron loss factor is calculated for each of the 18 single sheet tester samples.

(1) $$C=\frac{\mathrm{Iron}\;\mathrm{losses}\;\mathrm{of}\;12\;\mathrm{blanked}\;5\;\mathrm{mm}\;\mathrm{strips}}{\mathrm{Iron}\;\mathrm{losses}\;\mathrm{of}\;\mathrm{reference}\;\mathrm{sample}}$$

**Figure 6:**
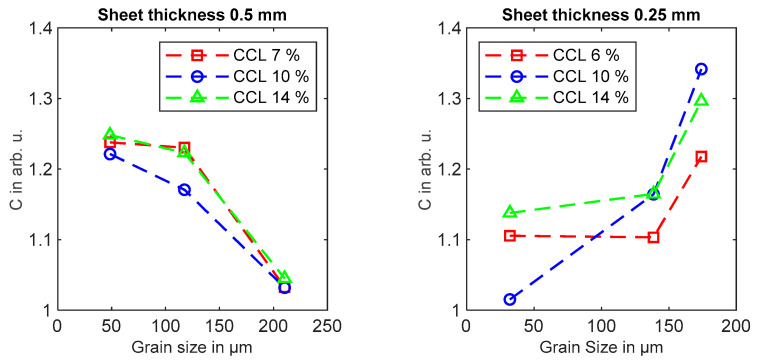
Iron loss factor C in dependence of the average grain size for sheet thicknesses of 0.25 mm or 0.5 mm and for cutting clearances of 6%, 7%, 10% and 14%.

In Figure 6, the iron loss factor C is displayed with respect to the average grain size. For the sheet thickness of 0.5 mm, the iron loss factor C is clearly reduced for larger grain sizes independent of the cutting clearance. For the sheet thickness of 0.25 mm, the correlation between the loss factor and grain size is inverse, with rising loss factors for increasing grain sizes. An influence of cutting clearance on the correlation between loss factor and grain size cannot be seen.

## 4. Discussion

### 4.1. Influence of Grain Size

Kals et al. conducted blanking experiments on CuZn15 sheets with sheet thicknesses between 0.25 mm and 1 mm, cutting clearances between 25 µm and 100 µm and grain sizes of 25 µm and 110 µm. They noticed an increase in shear strength for the combination of large grain size, small sheet thickness and small cutting clearance. This was attributed to an increased deformation resistance when the number of grains in the shear zone is reduced drastically as a consequence of miniaturization with respect to cutting clearance, punch diameter and sheet thickness [17].

The evaluated sheet thicknesses and cutting clearances in this work are very similar to [17], and the average grain sizes are even larger. Therefore, it can be concluded that size effects apply, which explains the observed rise in blanking work for increasing grain size, as displayed in Figure 4.

Weiss [11] evaluated the blanking of electrical steel sheets of sheet thicknesses between 0.30 mm and 0.65 mm with cutting clearances between 7 µm and 70 µm. The grain sizes of the examined materials lay between 60 to 119 µm (mean values of grain size measurements in rolling direction and transverse direction). The author noticed that the blanking work, as well as the blanking-related losses, increase with the cutting clearance, which was explained by the higher amounts of deformed material volume.

A direct correlation between cutting clearance and blanking work can also be seen in the results in Figure 4. However, there is no obvious influence of cutting clearance on the iron loss factor, which is in contradiction to [11,13,14], where rising cutting clearances lead to higher blanking-related losses.

On the other hand, Wang et al. [22] observed a minimum in iron loss deterioration for certain ‘optimum’ cutting clearances depending on grain size. They argue that smaller cutting clearances indeed reduce bending deformation, but also increase shear deformation at the same time. Scanning electron microscope measurements on the cut edges of blanked electrical steels confirmed that a reduction of the number of grains within the cutting clearance, e.g., by reduction of the cutting clearance, favors transgranular fracture [22]. Therefore, it seems likely that the beneficial influence of cutting clearance reduction on the iron loss factor is repealed by increasing transgranular fracture in this work.

More generally, there is no correlation between the blanking work and the iron loss factor. In fact, the iron loss factor C clearly decreases with increasing grain size for sheet thicknesses of 0.5 mm, while the blanking work is increased. This is in contradiction to the findings of Kuo et al., who noticed an elevated magnetic property deterioration after blanking with increasing grain size [13]. A possible explanation for the diverging results in this work is the higher ratio of grain size to sheet thickness. This means that blanking-induced dislocations can more easily reach a free surface and are less likely to pile up at grain boundaries. In this work and for 0.5 mm sheet thickness, the effect of dislocation annihilation at free surfaces seems to overcompensate plastic deformation and the beneficial effects of grain boundaries on residual stress influence described in [13]. Interestingly, the influence of grain size on the iron loss factor is inverted when looking at the 0.25 mm thick sheets, although dislocation annihilation should be encouraged even more due to the higher ratio of free surface to the overall material volume. Possibly, an increased ratio of transgranular fracture to intergranular fracture is the reason for the elevated iron loss factors when looking at the samples of 0.25 mm thickness. Transgranular fracture is very likely to happen with average grain sizes of up to 174 µm, which is more than half the sheet thickness.

### 4.2. Influence of Texture

According to Vollertsen [23], the annealing of sheet metals for the realization of different grain sizes with the intention to evaluate the grain size influence on sheet metal forming is questionable. This is because annealing also changes the texture of the material, which can affect the strength of the material in the specific forming process.

The influence of the cold rolling degree and the annealing temperature on the texture and grain size of the blanked steel sheets was evaluated by Wei et al. [18]. It was shown that the applied annealing temperature has a rather strong effect on texture compared to the cold rolling degree. Despite the fact that the annealing strategy was the same for both sheet thicknesses, the iron loss factors either increase or decrease, respectively, with increasing annealing temperature. This indicates that the influence of texture on blanking-related losses is rather small compared to the interplay of grain size and sheet thickness.

## 5. Conclusions

When blanking electrical steel of small sheet thickness and large grain size in combination with small cutting clearances, size effects occur. It appears likely that the rising shear strength with miniaturization that was observed by Kals et al. [17] is related to transgranular fracture. Large grain sizes can lead to lower blanking-related iron losses, which is attributed to dislocation annihilation. If miniaturization is too extreme, the influence of blanking on the iron losses becomes more pronounced with increasing grain size as a consequence of transgranular fracture. The blanking work does not necessarily correlate with blanking-related losses. This work shows that grain size has an influence on blanking-related iron losses. However, the findings of this work are only valid under the established experimental conditions of this work.

It seems feasible to develop steel grades with optimized grain sizes for the reduction of blanking-related losses, but further work needs to be done in order to make use of this discovery. Obviously, the type of fracture has a huge influence on the magnetic properties after blanking. The evaluation of the correlation between fracture model, dislocation density and magnetic properties in future work can potentially show ways for iron loss optimization in blanked parts.

## Figures and Tables

**Table 1:** Chemical composition of the investigated electrical steel measured by optical emission spectroscopy. S, N, P and C are either overestimated or below the detection limit of the method. Therefore, the maximum content based on supplier information (*) is provided.

ChemicalElement	Si	Al	Mn	S	N	P	C	Fe
wt.%	3.16	0.89	0.17	* 0.003	* 0.006	* 0.07	* 0.002	balance

**Table 2:** Overview of produced electrical steel sheets. Values in brackets represent the standard deviation.

Sheet Thickness in mm	Annealing Temperature in °C	Average Grain Size in The Mid Layer in µm	Average Grain Size in The Surface Layer in µm	Average Vickers Hardness
0.5	900	48 (±36)	37 (±22)	236 (±9)
0.5	1000	117 (±73)	87 (±55)	232 (±3)
0.5	1100	210 (±141)	179 (±115)	242 (±13)
0.25	900	32 (±19)	28 (±17)	211 (±8)
0.25	1000	139 (±82)	153 (±93)	221 (±7)
0.25	1100	174 (±96)	164 (±98)	225 (±5)

**Table 3:** Applied cutting clearances and the cutting clearance/sheet thickness ratios.

Sheet Thickness	Cutting Clearance in µm and % of the Sheet Thickness				
	15	25	35	50	70
0.25	6%	10%	14%	-	-
0.5	-	-	7%	10%	14%

## Data Availability

The data is available from the corresponding author upon reasonable request.
